# Proposal to create subspecies of *Rickettsia conorii *based on multi-locus sequence typing and an emended description of *Rickettsia conorii*

**DOI:** 10.1186/1471-2180-5-11

**Published:** 2005-03-14

**Authors:** Yong Zhu, Pierre-Edouard Fournier, Marina Eremeeva, Didier Raoult

**Affiliations:** 1 Unité des rickettsies, IFR 48, CNRS UMR 6020, Faculté de Médecine, Université de la Méditerranée, 27 Boulevard Jean Moulin, 13385 Marseille cedex 05, France; 2 Viral and Rickettsial Zoonoses Branch, Mail Stop G-13, Centers for Disease Control and Prevention, 1600 Clifton Road NE, Atlanta, GA 30333 USA

## Abstract

**Background:**

Rickettsiae closely related to the Malish strain, the reference *Rickettsia conorii *strain, include Indian tick typhus rickettsia (ITTR), Israeli spotted fever rickettsia (ISFR), and Astrakhan fever rickettsia (AFR). Although closely related genotypically, they are distinct serotypically. Using multilocus sequence typing (MLST), we have recently found that distinct serotypes may not always represent distinct species within the *Rickettsia *genus. We investigated the possibility of classifying rickettsiae closely related to *R. conorii *as *R. conorii *subspecies as proposed by the *ad hoc *committee on reconciliation of approaches to bacterial systematics. For this, we first estimated their genotypic variability by using MLST including the sequencing of 5 genes, of 31 rickettsial isolates closely related to *R. conorii *strain Malish, 1 ITTR isolate, 2 isolates and 3 tick amplicons of AFR, and 2 ISFR isolates. Then, we selected a representative of each MLST genotype and used multi-spacer typing (MST) and mouse serotyping to estimate their degree of taxonomic relatedness.

**Results:**

Among the 39 isolates or tick amplicons studied, four MLST genotypes were identified: i) the Malish type; ii) the ITTR type; iii) the AFR type; and iv) the ISFR type. Among these four MLST genotypes, the pairwise similarity in nucleotide sequence varied from 99.8 to 100%, 99.4 to 100%, 98.2 to 99.8%, 98.4 to 99.8%, and 99.2 to 99.9% for 16S rDNA, *glt*A, *omp*A, *omp*B, and *sca*4 genes, respectively. Representatives of the 4 MLST types were also classified within four types using MST genotyping as well as mouse serotyping.

**Conclusion:**

Although homogeneous genotypically, strains within the *R. conorii *species show MST genotypic, serotypic, and epidemio-clinical dissimilarities. We, therefore, propose to modify the nomenclature of the *R. conorii *species through the creation of subspecies. We propose the names *R. conorii *subsp. *conorii *subsp. nov. (type strain = Malish, ATCC VR-613), *R. conorii *subspecies *indica *subsp. nov. (type strain = ATCC VR-597), *R. conorii *subspecies *caspia *subsp. nov. (type strain = A-167), and *R. conorii *subspecies *israelensis *subsp. nov. (type strain = ISTT CDC1). The description of *R. conorii *is emended to accomodate the four subspecies.

## Background

**Figure 1:**
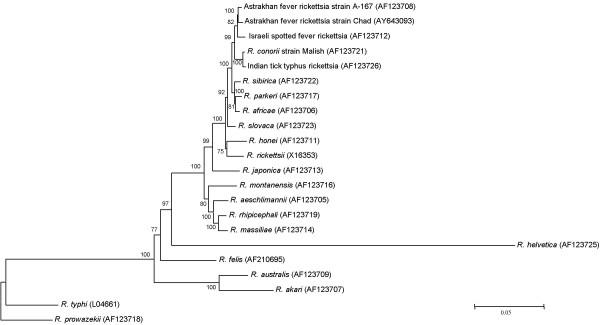
Unrooted phylogenetic tree derived from the comparison of sequences of the complete *glt*A gene from rickettsiae closely related to *R. conorii *and other validated *Rickettsia *species. The analysis used the Neighbor Joining method and the Kimura 2 parameter as described in the Methods section. Bootstrap values are indicated at the nodes of the phylogenetic tree. GenBank accession numbers are indicated in parentheses for each taxon.

Taxonomic classification of members of the order *Rickettsiales *was originally based on relatively few phenotypic criteria [1]. Over the past twenty years, the taxonomic classification of the Order *Rickettsiales *has undergone many changes. On the basis of 16S rDNA sequences, *Coxiella burnetii *was reclassified within the *Legionellaceae *[2], *Eperythrozoon *sp. within the *Mycoplasmataceae *[3], and *Rochalimaea *sp., *Grahamella *sp., and *Bartonella *sp. within the *Bartonellaceae*, closely related to *Brucella *sp [4,5]. Among the remaining members of the order *Rickettsiales*, the *Anaplasma-Ehrlichia *clade was recently reorganized into 4 genera [6]; and *R. tsutsugamushi *was reclassified into a new genus, *Orientia *[7]. Within the genus *Rickettsia*, since the pioneering work of Philip in 1978 [8], two rickettsial strains were considered as having different serotypes if they exhibited a specificity difference of ≥ 3 and some authors have considered this to be a useful guide to speciation [9,10]. However, opinions divide as to whether this holds true for rickettsial strains related to *Rickettsia conorii *(*R. conorii*) [11]. These include Indian tick typhus rickettsia (ITTR) [12], Israeli spotted fever rickettsia (ISFR) [13-15] and Astrakhan fever rickettsia (AFR) [16-19]. Phylogenetically, these rickettsiae constitute a homogeneous cluster supported by significant bootstrap values and are distinct from other *Rickettsia *species (Figure 1) [20-22]. Some authors have considered these rickettsial strains as different species and proposed the names *R. sharonii *and *R. caspii *for ISFR and AFR, respectively [23,24]. Other authors believe that they belong to a "*R. conorii *complex" [11,14,15,17,19].

These rickettsiae closely related to *R. conorii *have been described as the causative agents of Mediterranean spotted fever, Astrakhan fever, Israeli spotted fever, and Indian tick typhus in the Mediterranean basin and Africa, Southern Russia, Middle East, and India, respectively. These rickettsioses are transmitted to humans by *Rhipicephalus *ticks [1]. Furthermore, most of their clinical features overlap and are characterized by a febrile illness and a generalized maculopapular rash. However, an inoculation eschar is seldom present in Astrakhan fever and Israeli spotted fever but common in Mediterranean spotted fever (Table 1).

Recently, we have proposed gene sequence-based criteria for the identification of *Rickettsia *isolates at the genus, group and species levels and proposed criteria to define species [9]. Using these criteria, we have shown that ITTR, ISFR and AFR could not be considered as distinct species. Instead, they belonged to the *R. conorii *species [9]. However, these rickettsiae exhibit differentiable serotypes and cause diseases with distinct clinical features in defined geographic locations. The *ad hoc *committee on reconciliation of approaches to bacterial systematics [25] has proposed that even if related genetically, bacterial isolates within a given species could be considered as distinct subspecies if they differed phenotypically.

In this study, we undertook to examine whether phenotypic differences existed among various *R. conorii *isolates that would enable to classify them as subspecies. We estimated the degree of genotypic variabilities among 31 isolates of *R. conorii*, 1 isolate of ITTR, 2 isolates and 3 tick amplicons of AFR, and 2 isolates of ISFR using multi-locus sequence typing (MLST). We incorporated 16S rDNA and *glt*A genes, as well as 3 membrane-exposed protein-encoding genes *omp*A, *omp*B and *sca*4 (formerly gene D) [26,27] in MLST. To further characterize the specificities of distinct MLST types, we incorporated a prototype isolate from each of these into a multi-spacer typing (MST) assay, which we have previously demonstrated to be more discriminant than MLST at the strain level for *R. conorii *[28]. Furthermore, mouse serotypes were obtained for each of these MLST types.

## Results

### MLST-gene sequencing

All five genes studied were amplified from all 36 studied isolates and from 3 of 57 *Rhipicephalus pumilio *ticks tested. All negative controls remained negative. Four MLST types were identified. Within the first type, which we named Malish type, all *R. conorii *isolates exhibited identical sequences for each of the five genes tested except isolates Moroccan and M1. These latter isolates showed deletions of 24 and 156 bp, respectively, at the 5'-end of the *omp*A gene. The second type, the ITTR type, was made of ITTR. This exhibited unique nucleotide substitutions in *omp*A, *omp*B and *sca*4 genes. Within the AFR type, all isolates and tick amplicons tested showed identical sequences of the five genes studied except the Chad isolate which showed subtle nucleotide substitutions in each of them. In this isolate, the pairwise nucleotide similarity with AFR for 16S rDNA, *glt*A, *omp*A, *omp*B and *sca*4 genes was 99.7, 99.5, 99.5, 99.2, and 99.8 %, respectively. The fourth type, ISFR type, which included both ISFR isolates, showed the same nucleotide substitutions in each of the five genes tested. This differentiated them from other rickettsiae. As representative strains for each MLST genotype, we have chosen *R. conorii *isolate Malish, ITTR isolate Indian, AFR isolate A-167, and ISFR isolate ISTT CDC1.

Among representatives of the four MLST genotypes, the degree of pairwise nucleotide similarity ranged from 99.8 % between AFR and ISFR to 100 % between *R. conorii *and ITTR for the 16S rDNA gene (Table 3). For *glt*A, such similarity ranged from 99.5 % between Malish (or ITTR) and ISFR to 100 % between *R. conorii *and ITTR. For the 5' end of *omp*A, the degree of pairwise sequence similarity ranged from 98.1 between AFR and ITTR to 99.8% between *R. conorii *and ITTR. This pairwise similarity ranged from 98.5 between ITTR and ISFR to 99.8% between *R. conorii *and ITTR for *omp*B. For *sca*4, the pairwise sequence similarity varied from 99.2 between ITTR and ISFR to 99.9% between *R. conorii *and ITTR.

**Figure 2:**
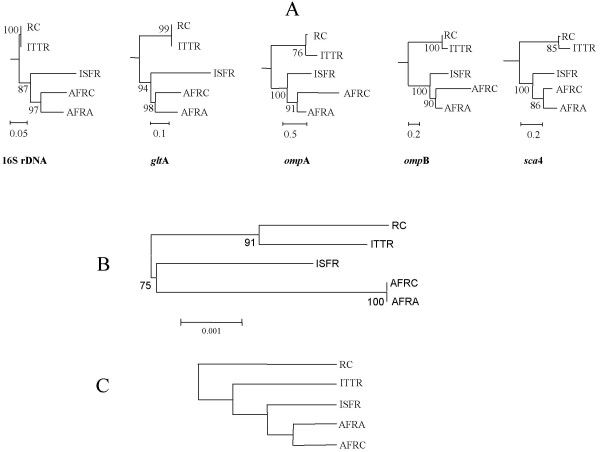
A = Unrooted phylogenetic trees derived from the comparison of sequences of the 16S rDNA, *glt*A, *omp*A, *omp*B and *sca*4 genes using the Neighbor Joining method (A). Bootstraps values are indicated at the nodes of the phylogenetic tree. The scale bars represent the percentage of nucleotide differences. RC = *R. conorii*; ITTR = Indian tick typhus rickettsia; AFRA = AFR isolate A-167; AFRC = AFR isolate Chad; ISFR = Israeli spotted fever rickettsia. B = Unrooted phylogenetic trees derived from the comparison of concatenated sequences of the *dks*A / *xer*C, *mpp*A / *pur*C, and *rpm*E / tRNA-fMet intergenic spacers using the Neighbor Joining method (B). Bootstraps values are indicated at the nodes of the phylogenetic tree. The scale bars represent the percentage of nucleotide differences. RC = *R. conorii*; ITTR = Indian tick typhus rickettsia; AFRA = AFR isolate A-167; AFRC = AFR isolate Chad; ISFR = Israeli spotted fever rickettsia. C = Taxonomic dendrogram of rickettsiae closely related to *Rickettsia conorii *obtained by using the unweighted pair group method based on reactivity of mouse antisera to all tested rickettsiae. RC = *R. conorii*; ITTR = Indian tick typhus rickettsia; AFRA = AFR isolate A-167; AFRC = AFR isolate Chad; ISFR = Israeli spotted fever rickettsia.

The dendrograms obtained using the three different tree building analysis methods showed similar organization for the five rickettsiae studied. We identified two monophyletic groups with the trees so constructed. One included *R. conorii *and ITTR and the other, ISFR and AFR (Figure 2A). The clustering of these rickettsiae was supported by high bootstrap values. Both AFR isolates were grouped together for each of the five genes tested.

### MST genotyping

ITTR showed unique 232-bp *dks*A / *xer*C (type P, GenBank accession number AY836513) and 153-bp *mpp*A / *pur*C (type F, AY836517) spacer sequences. However, it also showed a 200-bp *rpm*E / tRNA-fMet spacer sequence identical to that of *R. conorii *type B (AY345092); ISFR had unique 232-bp *dks*A / *xer*C (type Q, AY836510), 153-bp *mpp*A / *pur*C (type G, AY836514), and 200-bp *rpm*E / tRNA-fMet (type C, AY836518) spacer sequences; AFR isolates A-167 and Chad showed unique *dks*A / *xer*C of 232- (type R, AY836511) and 167-bp (type S, AY836512), respectively. They also showed identical 153-bp *mpp*A / *pur*C (type H, AY836516) and 200-bp *rpm*E / tRNA-fMet (type D, AY836519) spacer sequences that were different from those of other strains. When combining the genotypes obtained from each of the three spacers into a MST genotype, each of the four rickettsiae showed a distinct MST genotype. The MST genotype so obtained was different from that of *R. conorii*. However, on pairwise comparisons of the four strains, both AFR strains showed similar spacer types. The phylogenetic tree inferred from the concatenated spacer sequences showed an organization similar to that obtained from MLST when Malish strain was taken as *R. conorii *type strain (Figure 2B). However, the node where ISFR branched was not supported by a significant bootstrap value.

### Microimmunofluorescence (MIF) serotyping

When injected into mice, all five rickettsiae tested provoked antibody responses that were detectable by MIF. The endpoint titers and specificity differences (SPDs) were calculated as described in the Methods section. These values are presented in Table 4. The antibody titres obtained in homologous sera were at least one-fold higher than those observed in heterologous sera. The SPDs for heterologous sera varied between 1 and 5. Four serotypes were identified. These included *R. conorii*, ITTR, both AFR isolates, and ISFR. The dendrogram inferred from the matrix based on SPDs (Figure 2C) showed that ITTR occupied a position that slightly differed from its phylogenetic organization. Instead of forming a cluster with *R. conorii*, it occupied an intermediate position between *R. conorii *and ISFR (Figures 2A and 2B).

## Discussion

We propose to create subspecies within the *R. conorii *species to accommodate both the genotypic homogeneity and the serotypic, MST genotypic, geographic and pathogenic diversities of rickettsia strains within this species.

Defining a species within the *Rickettsia *genus has long been difficult but rickettsiologists have agreed to use mouse serotyping as one criterion [8]. This classification scheme served as a basis for the development of genotypic criteria at the species level [9]. However, confusion still exists about the taxonomic status of rickettsiae. This is especially the case for rickettsiae which are closely related to classic *R. conorii *isolates. Philip *et al*. [8], using mouse serotyping, have demonstrated that *R. conorii *isolates Malish, Moroccan and Kenya were closely related antigenically to ITTR and that they belonged to a single serotype. Using complement fixation, Bozeman *et al*. [29] were unable to distinguish ITTR from *R. conorii *isolates. In contrast, others, using mouse polyclonal antibodies [14], and monoclonal antibodies [11], have demonstrated substantial differences between ITTR and *R. conorii *isolates. ISFR and AFR could not be distinguished using PCR-RFLP [17] until *omp*A was used as target gene [30]. In 1993, we have demonstrated that AFR differed from both ISFR and *R. conorii *in terms of SDS-PAGE mobility and PFGE profiles [19]. Although they are difficult to distinguish serologically, the cross-reacting epitopes they share are located on antigenic proteins that differ in SDS-PAGE mobility and hence in molecular weight [19]. In 1995, Walker *et al.*, using serotyping, Western bloting, monoclonal antibody reactivity and PCR amplification of the tandem repeats within *omp*A, concluded that ISFR belonged to the *R. conorii *species [15]. In 1998, Dasch and Jackson differentiated *R. conorii *from ISFR using PCR-RFLP [23]. Recently, using a combination of genotypic criteria, we have demonstrated that the genotypic differences among ITTR, AFR and ISFR were too subtle to classify them as new species [9]. Instead, they belonged to the *R. conorii *species.

In the present study, we have demonstrated, using MLST, that rickettsiae closely related to *R. conorii *isolate Malish are very homogeneous. Except isolates Moroccan and M1 which showed one *omp*A deletion of 24 and 156 bp, respectively, all studied *R. conorii *isolates had identical sequences for the five genes studied and thus constituted a reliable taxon. The ITTR isolate showed unique sequences of *omp*A, *omp*B and *sca*4 genes. Among isolates and tick amplicons of AFR, the degree of MLST sequence similarity was of 100% except for the Chad isolate which is genotypically slightly different from AFR isolate A-167 [31]. In another study, we have found identical *omp*A sequences in four tick amplicons from Kosovo and AFR isolate A-167 [32]. We have also observed serologic, antigenic and genetic homogeneities among two tick and one human AFR isolates from Astrakhan [19]. Thus, except the Chad isolate which was slightly different genotypically, AFR, with three strains and ten tick amplicons studied, appeared to form a homogeneous taxon. The two isolates of ISFR tested showed 100% pairwise nucleotide similarity in all five genes studied. As we have compared only two isolates, one may argue that this may not be representative of the diversity of *omp*A in ISFR. However, Walker *et al*. found four human ISFR isolates from Israel to be highly homogeneous antigenically as well as genetically [15]. Bacellar *et al. *described three human isolates of ISFR from Portugal that showed 100% similarity in 16S rDNA, *glt*A and *omp*A sequence with those of ISFR isolate ISTTCDC1 [33]. Giammanco *et al*. identified a tick rickettsial isolate in Italy that had 100% *omp*A sequence similarity with ISFR isolate ISTTCDC1 [34]. Therefore, it appeared that our ISFR isolates were representatives of the same homogeneous taxon.

In the first part of the study, we have identified four MLST genotypes which were subjected to further evaluation. Among representatives of these MLST genotypes, the minimal pairwise similarity in the nucleotide sequences of 16S rDNA, *glt*A, *omp*A, *omp*B, and *sca*4 genes between the rickettsial isolates tested and *R. conorii*, was 99.8%, 99.4%, 98.3%, 98.5%, and 99.3%, respectively. Thus, All four isolates tested fulfilled the criteria to belong to the *R. conorii *species [9].

Phylogenetically, the members of the *R. conorii *complex have been demonstrated to form a stable group distinct from other *Rickettsia *species (Figure 1) [20-22]. We found that within this cluster they consistently formed two phylogenetic clusters (Figure 2A). One of these clusters included *R. conorii *and ITTR, and the other, AFR isolates and ISFR. Using MST, we identified five genotypes. However, using pairwise comparison of spacer sequences, we identified only four genotypes similar to those obtained by MLST. MST-based phylogeny also demonstrated a similar pattern (Figure 2B). Serotypically, we could identify four serotypes which, according to the definition of species proposed by Philip [8], would classify them as different species: 1) *R. conorii *isolate Malish; 2) ITTR; 3) AFR; and 4) ISFR. These four serotypes also showed distinct MST genotypes.

We demonstrated a discrepancy between the genotypic homogeneity of these rickettsiae, which classified them within the *R. conorii *species [9], and their MST genotypic, serotypic, geographic, and pathogenic heterogeneity. For ITTR, an additional discrepancy was observed between its phylogenetic and serotypic classifications (Figures 2A and 2B versus 2C). Such findings are in accordance with those of Walker *et al*. who had shown substantial differences between ITTR and *R. conorii *isolates [11]. We also agree with these authors who hypothesized that all members of the "so-called" *R. conorii *complex belonged to the *R. conorii *species [15].

## Conclusion

We have demonstrated that *R. conorii *and closely related strains exhibit a high level of conservation in nucleotide sequence despite genotypic specificities, and phenotypic specificities including serotyping, epidemiological and clinical characteristics. Therefore, according to the accepted criteria of the *ad hoc *committee on reconciliation of approaches to bacterial systematics which allow the creation of subspecies for genetically-close isolates which express phenotypic differences [25], we are justified in creating new subspecies for these organisms.

### Emendation of the description of *Rickettsia conorii*

*Rickettsia conorii *(Brumpt 1932). The characteristics of this taxon are similar to those described by Weiss and Moulder for the species [1]. The species contains four subspecies. The minimal degree of pairwise nucleotide similarity of the *R. conorii *species is of 99.8 % for 16S rDNA, 99.4 % for *glt*A, 98.3 % for the 5' end of *omp*A, 98.6 % for *omp*B, and 99.3 % for *sca*4, by comparison with isolate Malish.

### Description of *Rickettsia conorii *subsp. *conorii *subsp. nov

*Rickettsia conorii *subsp. *conorii *(co.no'ri.i N. L. gen. n. *conorii*, of Conor, in honor of A. Conor who, in collaboration with A. Bruch, provided the first description of fièvre boutonneuse in 1910 [35]). The characteristics are the same as those of the species. Transmitted to humans through the bite of *Rhipicephalus sanguineus *ticks. Grows in Vero cells at 32°C in antibiotic-free Minimal Essential Medium supplemented with 2 % fetal calf serum and 2 mg/ml L-glutamine.

The type strain is Malish, ATCC VR-613, which was isolated in South Africa by J.H.S. Gear in 1946. This type is the most common and representative of the isolates of *R. conorii *subsp. *conorii *subsp. nov. The nucleotide sequence of the 16S rRNA, *glt*A, *omp*A, *omp*B, and *sca*4 genes have been deposited in the GenBank database under the accession numbers, AF541999, U59730, U43806 [30], AF123721 [22], and AF163008 [20], respectively. To be classified as *R. conorii *subsp. *conorii *subsp. nov., a rickettsial isolate should exhibit a minimal pairwise nucleotide sequence similarity of 100%, 100%, 99.9%, 99.9%, and 100% for the 16S rDNA, *glt*A, *omp*A, *omp*B, and *sca*4 genes, respectively, with those of *R. conorii *isolate Malish, ATCC VR-613. *R. conorii *subsp. *conorii *subsp nov. strain Malish has also been deposited in the official collection of the WHO Collaborative Center for the Diagnosis and Study of Rickettsioses in Marseille, France.

### Description of *Rickettsia conorii *subsp. *indica *subsp. nov

*Rickettsia conorii *subsp. *indica *(in'di.ca N. L. gen. n. *indica*, from India, where the *Rhipicephalus sanguineus *tick providing the first isolate was collected). The characteristics are the same as those of the species. Transmitted to humans through the bite of *Rhipicephalus sanguineus *ticks. Grows in Vero cells at 32°C in antibiotic-free Minimal Essential Medium supplemented with 2 % fetal calf serum and 2 mg/ml L-glutamine.

The type strain is Indian tick typhus, ATCC VR-597, which was isolated from a *Rhipicephalus sanguineus *tick collected in India by C.B. Philip in 1950. The nucleotide sequence of the 16S rRNA, *glt*A, *omp*A, *omp*B, and *sca*4 genes have been deposited in the GenBank database under the accession numbers L36107 [36], U59730 [37], U43794 [30], AF123726 [22], and AF163005 [20], respectively. To be classified as *R. conorii *subsp. *indica *subsp. nov., a rickettsial isolate should exhibit a minimal pairwise nucleotide sequence similarity of 100%, 100%, 99.9%, 99.9%, and 100% for the 16S rDNA, *glt*A,*omp*A, *omp*B, and *sca*4 genes, respectively, with those of *R. conorii *subsp. *indica *subsp. nov., isolate Indian tick typhus, ATCC VR-597. *R. conorii *subsp.*conorii *subsp nov. isolate Indian has also been deposited in the official collections of the WHO Collaborative Center for the Diagnosis and Study of Rickettsioses in Marseille, France; of the WHO and National Reference Collection at the Gamaleya Research Institute, Moscow, Russia, and of the WHO Collaborating Reference Center for Rickettsial and Bartonella-associated Diseases, CDC, Atlanta, USA.

### Description of *Rickettsia conorii *subsp. *caspia *subsp. nov

*Rickettsia conorii *subsp. *caspia *(cas' pi.a N. L. fem.adj. *caspia*, from Mare Caspium, the Latin name of the Caspian sea where the disease caused by this rickettsia is endemic). The characteristics are the same as those of the species. Transmitted to humans through the bite of *Rhipicephalus sanguineus *and *R. pumilio *ticks. Grows in Vero cells at 32°C in antibiotic-free Minimal Essential Medium supplemented with 2 % fetal calf serum and 2 mg/ml L-glutamine.

The type strain is A-167, which was isolated from a *Rhipicephalus pumilio *tick collected in Astrakhan in 1992. The nucleotide sequence of the 16S rRNA, *glt*A, *omp*A, *omp*B, and *sca*4 genes have been deposited in the GenBank database under the accession numbers L36100 [36], U59728 [37], U43791 [30], AF123708 [22], and AF163007 [20], respectively. The nucleotide sequence of the 16S rRNA, *glt*A, *omp*A, *omp*B, and *sca*4 genes of AFR isolate Chad have been deposited in the GenBank database under the accession numbers AF510102 [31], AF510103 [31], AY112668 [31], AY643093, and AY643092, respectively. To be classified as *R. conorii *subsp. *caspia *subsp. nov., a rickettsial strain should exhibit a minimal pairwise nucleotide sequence similarity of 99.7%, 99.6%, 99.5%, 98.9%, and 99.6% for the 16S rDNA, *glt*A, *omp*A, *omp*B, and *sca*4 genes, respectively, with those of *R. conorii *subsp. *caspia *subsp. nov., isolate A-167. *R. conorii *subsp. *caspia *subsp nov isolate A-167 has been deposited in the official collections of the WHO Collaborative Center for the Diagnosis and Study of Rickettsioses in Marseille, France, and of the WHO Collaborating Reference Center for Rickettsial and Bartonella-associated Diseases, CDC, Atlanta, USA.

### Description of *Rickettsia conorii *subsp. *israelensis *subsp. nov

*Rickettsia conorii *subsp. *israelensis *(is.ra. el en' sis. N. L. gen. n. *israelensis*, from Israel, where the *Rhipicephalus sanguineus *tick providing the first isolate was collected). The characteristics are the same as those of the species. Transmitted to humans through the bite of *Rhipicephalus sanguineus *ticks. Grows in Vero cells at 32°C in antibiotic-free Minimal Essential Medium supplemented with 2 % fetal calf serum and 2 mg/ml L-glutamine.

The type strain is ISTTCDC1, which was isolated from a *Rhipicephalus sanguineus *tick collected in Israel in 1974. The nucleotide sequence of the 16S rRNA, *glt*A, *omp*A, *omp*B, *sca*4 genes have been deposited in the GenBank database under the accession numbers L36223 [36], U59727 [37], U43797 [30], AF123712 [22], and AF155058 [20], respectively. To be classified as *R. conorii *subsp. *israelensis *subsp. nov., a rickettsial isolate should exhibit a minimal pairwise nucleotide sequence similarity of 100%, 99.7%, 98.9%, 99.5%, and 99.8%, for the 16S rDNA, *glt*A, *omp*A, *omp*B, and *sca*4 genes, respectively, with those of *R. conorii *subsp. *israelensis *subsp. nov., isolate ISTTCDC1. *R. conorii *subsp. *israelensis *subsp. Nov. isolate ISTTCDC1 has been deposited in the official collections of the WHO Collaborative Center for the Diagnosis and Study of Rickettsioses in Marseille, France, and of the WHO Collaborating Reference Center for Rickettsial and Bartonella-associated Diseases, CDC, Atlanta, USA.

## Methods

### Rickettsial strains

All rickettsial strains tested and their original sources are listed in Table 2. Rickettsial isolates for which sequences were not available were grown in Vero cell monolayers at 32°C in 150 cm^2^-cell culture flasks as previously described [37]. When Gimenez staining demonstrated the cells have been infected heavily, they were harvested using sterile glass beads and centrifuged at 12,000 *g *for 10 min, resuspended in fresh medium and stored at -70°C until purification of nucleic acid was carried out. All rickettsial stocks were tested and shown to be free of *Mycoplasma *sp using a 16S rDNA-based PCR assay as previously described [38].

### Nucleic acid purification, PCR amplification and sequencing

When available, we used gene sequences present in GenBank. The accession numbers for these sequences are reported at the end of the manuscript. Missing sequences were obtained in this study. Genomic DNA was extracted from 200 μl of rickettsial suspension and from 57 *Rhipicephalus pumilio *ticks collected in Astrakhan [19] using the QIAamp Tissue Kit (Qiagen, Hilden, Germany) according to the manufacturer's instructions. For MLST, we used the previously described primers and PCR conditions to amplify 1,424-bp, 1,134-bp, 590-bp, 4,890-bp, and 3,028-bp fragments of the 16S rDNA [36], *gltA *[37], *ompA *[30], *ompB *[22] and *sca4 *[20] genes, respectively, of 29 of 36 rickettsial isolates tested for which no sequences were available in GenBank, and 57 *R. pumilio *ticks. In addition, we amplified and sequenced the *omp*B and *sca*4 genes of AFR isolate Chad which had not been determined previously. Primers were purchased from Eurogentec (Seraing, Belgium). PCR reactions were carried out in a Peltier model PTC-200 thermal cycler (MJ Research, Watertown, MA). Each PCR included a negative (distilled water) and a positive control (DNA extracted from *R. montanensis *isolate 2–4–6 using the QIAamp Tissue Kit [Qiagen]). PCR products were visualized after electrophoresis on a 1% agarose gel. Each amplicon was purified for sequencing using the QIAquick Spin PCR Purification Kit (Qiagen) according to the manufacturer's instructions. Sequencing reactions were carried out using PCR primers and the D-rhodamine terminator cycle DNA sequencing Kit (Applied Biosystems, Foster City, California) according to the manufacturer' instructions. Sequencing reaction products were resolved by electrophoresis with an ABI Prism 3100 Automated Sequencer (Applied Bioystems). Each base position was established twice in both the forward and reverse directions.

For each gene, the pairwise sequence similarity among studied isolates and tick amplicons was estimated using the multisequence alignment program CLUSTAL W [39]. Each isolate or tick amplicon exhibiting specific nucleotide substitutions within one or more of the five genes studied was considered a different MLST genotype. Then, we selected a representative isolate of each MLST genotype for further tests. When a MLST genotype incorporated more than one isolate, we selected the type strain or the first strain described in the literature as representative strain. In addition to AFR isolate A-167, we incorporated AFR isolate Chad in the phylogenetic study and the mouse immunization and MST assays because MLST sequence differences between both isolates were due to nucleotide substitutions as well as deletions.

Distance matrices generated by DNADIST were determined under the assumptions of Kimura and were used to infer dendrograms by the neighbor-joining method [40] using the PHYLIP version 3.4 software package [41]. Dendrograms were also constructed by data processing with the maximum-likehood and parsimony methods (DNAML and DNAPARS, respectively, in PHYLIP). A bootstrap analysis based on 100 randomly generated trees using SEQBOOT and CONSENSE in PHYLIP was performed to estimate the node reliability of the trees obtained by three phylogenetic methods [42].

### Multi-spacer typing (MST)

We amplified and sequenced the *dks*A / *xer*C, *mpp*A / *pur*C, and *rpm*E / tRNA-fMet intergenic spacers for each of *R. conorii *isolate Malish, ITTR, ISFR, and AFR isolates A-167 and Chad. Primers and methods were those described previously [28]. Subsequently, spacer sequences were concatenated and used to infer the phylogenetic relationships among strains tested using the neighbor-joining method [40] as described above.

### Mouse immunization

For each *R. conorii *isolate Malish, ITTR, ISFR, and AFR isolates A-167 and Chad, five BALB/c mice were inoculated intravenously (tail vein) with purified bacterial suspension (0.1 ml containing 10^4 ^bacteria). The concentration of rickettsial suspensions was estimated using a plaque assay as previously described [43]. On day 7, mice were boosted with a similar inoculum. On day 10, mice were anesthetized and exsanguinated by cardiac puncture [8]. Sera from each group of five mice were pooled and stored at -20°C until used for microimmunofluorescence (MIF) serotyping.

### MIF serotyping

The MIF test was performed and the SPD was calculated using the method of Philip et al. [8]. Each of the five rickettsiae tested was compared with each other. The highest serum dilutions giving positive reactions were recorded as endpoint titer: SPD = (Aa + Bb) - (Ab + Ba), where Aa or Bb is -log2 of the endpoint titer between serum A or B and homologous antigen a or b and Ab or Ba is -log2 of the endpoint titer of serum A or B against heterologous antigen b or a. If the SPD was less than 3, the two isolates were assumed to belong to the same serotype. If the SPD was ≥ 3, the isolates were assumed to belong to different serotypes. The SPDs were used to construct a matrix, and then a dendrogram was constructed from the matrix by the unweighted pair group method with arithmetic mean (UPGMA) available in the MEGA2 software package [44].

## Authors' contributions

The individual parts of the work presented in the paper were conducted as follows: YZ and PEF carried out the molecular genetic studies, analyzed the sequences and drafted the manuscript. ME participated in the design of the study and helped to draft the manuscript. DR conceived the study, and participated in its design and coordination and helped to draft the manuscript. All authors read and approved the final manuscript.

## Figures and Tables

**Table 1:** Ecological, epidemiological and clinical characteristics of diseases associated with different members of the *R. conorii *complex

**Rickettsia**	**Vector tick**	**Geographic repartition**	**Human disease name**	**Fever (%)**	**Inoculation eschar (%)**	**Cutaneous rash (%)**	**Fatal forms**	**Reference**
*R. conorii*, isolates Malish, Moroccan *R. conorii*, isolate Kenyan tick typhus rickettsia	*Rhipicephalus *sp., *Haemaphysalis leachii*	Mediterranean area, Africa	Mediterranean spotted fever, Kenyan tick typhus, South African tick bite fever	100	72	97	Yes	[45,46]
Indian tick typhus rickettsia	*Rhipicephalus sanguineus*, *Boophilus microplus*,* Haemaphysalis leachii*	India, Pakistan	Indian tick typhus	100	0	100	No	[47]
Astrakhan spotted fever rickettsia	*Rhipicephalus sanguineus, R. pumilio*	Astrakhan region, Chad, Kosovo	Astrakhan fever	100	23–28	100	No	[16-18,31,32]
Israeli tick typhus rickettsia	*Rhipicephalus sanguineus*	Israel, Portugal	Israeli spotted fever	100	0	100	Yes	[48]

**Table 2:** Rickettsial isolates included in the study

**Strain (ATCC number)**	**Original source**	**Geographical origin**	**Human disease**	Passage history*	**Strain provided by**	**Reference**
***R. conorii***						
Moroccan (VR-141)	Unknown	Morocco	Mediterranean spotted fever	118	ATCC	[49]
Malish (VR-613)	Unknown	South Africa	Mediterranean spotted fever	67	ATCC	Gear
Kenya	*Haemaphysalis leachii*	Kenya	Kenya tick typhus	17	G. Dasch ¶	[49]
M1	*Rhipicephalus sanguineus*	Georgia, former Soviet Union		12	Gamaleya Institute, Moscow	[50]
Zim 1	*Rhipicephalus simus*	Zimbabwe		10	P.J. Kelly **	[10]
Zim C	*Haemaphysalis leachii*	Zimbabwe		8	P.J. Kelly **	[51]
URRCSpain3	Human	Spain	Mediterranean spotted fever	20		PS ‡
16-B	Human	Spain	Mediterranean spotted fever	20	†	[52]
Spain96	Human	Spain	Mediterranean spotted fever	5	N. Cardenosa ¶¶	[53]
Portugal454	Human	Portugal	Mediterranean spotted fever	4	F. Bacellar † †	PS ‡
Portugal821	Human	Portugal	Mediterranean spotted fever	4	F. Bacellar † †	PS ‡
Portugal162	Human	Portugal	Mediterranean spotted fever	4	F. Bacellar † †	PS ‡
Portugal5	Human	Portugal	Mediterranean spotted fever	4		PS ‡
URRCFrance1	Human	France	Mediterranean spotted fever	4	†	PS ‡
URRCFrance2	Human	France	Mediterranean spotted fever	4	†	PS ‡
URRCFranceFEe4	Human	France	Mediterranean spotted fever	4	†	PS ‡
URRCFranceFEe5	Human	France	Mediterranean spotted fever	4	†	PS ‡
URRCFranceFEe6	Human	France	Mediterranean spotted fever	4	†	PS ‡
URRCFranceE7	Human	France	Mediterranean spotted fever	4	†	PS ‡
URRCFranceFEe8	Human	France	Mediterranean spotted fever	4	†	PS ‡
URRCFranceF9	Human	France	Mediterranean spotted fever	4	†	PS ‡
URRCFrance10	Human	France	Mediterranean spotted fever	4	†	PS ‡
URRCFranceFE11	Human	France	Mediterranean spotted fever	4	†	PS ‡
URRCFranceFE17	Human	France	Mediterranean spotted fever	4	†	PS ‡
URRCAlgeria18	Human	Algeria	Mediterranean spotted fever	4	†	PS ‡
URRCFranceFEe25	Human	France	Mediterranean spotted fever	4	†	PS ‡
URRCTunisia28	Human	Tunisia	Mediterranean spotted fever	4	†	PS ‡
URRCCroatia29	Human	Croatia	Mediterranean spotted fever	4	†	PS ‡
URRCFranceFEe31	Human	France	Mediterranean spotted fever	4	†	PS ‡
URRCFranceFEe32	Human	France	Mediterranean spotted fever	4	†	PS ‡
AH	Human	France	Mediterranean spotted fever	7	†	PS ‡
						
**Indian tick typhus rickettsia**						
Indian (VR-597)	*Rhipicephalus sanguineus*	India	Indian tick typhus	20	ATCC	[12]
**Astrakhan fever rickettsia**						
A-167	*Rhipicephalus pumilio*	Astrakhan region, former Soviet Union	Astrakhan fever	15	†	[19]
Chad	Human	Chad	Unnamed spotted fever	4	†	[31]
						
**Israeli spotted fever rickettsia**						
ISTT CDC1	*Rhipicephalus sanguineus*	Israel	Israeli spotted fever	35	G. Dasch ¶	[14]
P426	Human	Portugal	Unnamed spotted fever	6	F. Bacellar † †	[33]

* The passage history refers to the number of passages of the strain on Vero cells (ATCC CRL-1587)

¶ Centers for Disease Control, Atlanta, GA, U.S.A.

† Isolated in our laboratory

‡ PS = present study

** Veterinary Teaching Hospital, Massey University, Palmerston North, New Zealand

¶¶Programa Asistencial de Malalties Infeccioses I Seccio de Microbiologia, Corporacio Sanitaria Parc Tauli, Sabadell, Barcelona, Spain

† † Centro de Estudos de Vectores e Doenças Infecciosas, Instituto Nacional de Saúde Dr. Ricardo Jorge, Aguas de Moura, Portugal

**Table 3:** Pairwise nucleotide sequence similarities among members of the *R. conorii *complex

	% Similarity		
	
Rickettsia and gene	ITTR	AFR isolate A-167	ISFR
***R. conorii***			
16S rDNA	100	99.9	99.8
*glt*A	100	99.5	99.3
*omp*A	99.8	98.3	98.3
*omp*B	99.8	98.7	98.6
*sca*4	99.9	99.4	99.3

**ITTR**			
16S rDNA		99.9	99.8
*glt*A		99.5	99.3
*omp*A		98.1	98.1
*omp*B		98.6	98.5
*sca*4		99.3	99.2

**AFR isolate A-167**			
16S rDNA			99.8
*glt*A			99.7
*omp*A			98.6
*omp*B			99.4
*sca*4			99.7

**Table 4:** MIF antibody titers and SPDs from reciprocal cross-reactions of mouse antisera to the members of the *Rickettsia conorii *complex.

	MIF antibody titer (SPD) of mouse antisera to *:				
Antigens	*R. conorii*	ITTR	AFR isolate A-167	AFR isolate Chad	ISFR

*R. conorii*	1,024	256 (4)	256 (5)	256 (5)	256 (4)
ITTR	512 (4)	2,048	512 (4)	256 (5)	256 (5)
AFR isolate A-167	128 (5)	256 (4)	1,024	512 (2)	256 (4)
AFR isolate Chad	128 (5)	256 (5)	512 (2)	1,024	256 (4)
ISFR	128 (4)	128 (5)	128 (4)	128 (4)	512

* Titers are the reciprocals of the highest dilution of antisera that gave a positive reaction. SPDs are calculated according to the formula given in the Materials and Methods section.
